# Differences in Noradrenaline Receptor Expression Across Different Neuronal Subtypes in Macaque Frontal Eye Field

**DOI:** 10.3389/fnana.2020.574130

**Published:** 2020-11-26

**Authors:** Max Lee, Adrienne Mueller, Tirin Moore

**Affiliations:** ^1^Department of Neurobiology, Howard Hughes Medical Institute, Stanford University School of Medicine, Stanford, CA, United States; ^2^Department of Neurobiology, Stanford University, Stanford, CA, United States

**Keywords:** neuromodulators, working memory, attention, microcircuit, macaque, immunofluorescence

## Abstract

Cognitive functions such as attention and working memory are modulated by noradrenaline receptors in the prefrontal cortex (PFC). The frontal eye field (FEF) has been shown to play an important role in visual spatial attention. However, little is known about the underlying circuitry. The aim of this study was to characterize the expression of noradrenaline receptors on different pyramidal neuron and inhibitory interneuron subtypes in macaque FEF. Using immunofluorescence, we found broad expression of noradrenaline receptors across all layers of the FEF. Differences in the expression of different noradrenaline receptors were observed across different inhibitory interneuron subtypes. No significant differences were observed in the expression of noradrenaline receptors across different pyramidal neuron subtypes. However, we found that putative long-range projecting pyramidal neurons expressed all noradrenaline receptor subtypes at a much higher proportion than any of the other neuronal subtypes. Nearly all long-range projecting pyramidal neurons expressed all types of noradrenaline receptor, suggesting that there is no receptor-specific machinery acting on these long-range projecting pyramidal neurons. This pattern of expression among long-range projecting pyramidal neurons suggests a mechanism by which noradrenergic modulation of FEF activity influences attention and working memory.

## Introduction

Previous research has established the importance of adrenergic signaling in the prefrontal cortex (PFC) for cognitive functions such as attention and working memory (Aoki et al., 1998a,b; Xing et al., 2016). Furthermore, adrenergic signaling plays a role in numerous neuropsychiatric diseases including attention-deficit hyperactivity disorder (ADHD), Alzheimer’s Disease, and Parkinson’s Disease (Borodovitsyna et al., 2017). Studies in human and animal models have established an important role of the frontal eye field (FEF), an oculomotor area of the PFC, in the control of visuo-spatial attention (Moore and Zirnsak, 2017). Neurons in the FEF of human subjects appears to be modulated by noradrenaline (Grefkes et al., 2010). Yet, the influence of adrenergic input on FEF circuitry is poorly understood.

Adrenergic input to the PFC has been shown to stem primarily from the locus coeruleus (Porrino and Goldman-Rakic, 1982; Van Gaalen et al., 1997). Noradrenaline receptors are classified into three classes: α1 adrenergic receptors (α1A, α1B, α1D), α2 adrenergic receptors (α2A, α2B, α2C), and β adrenergic receptors (β1, β2, β3; Ahlquist, 1948, reviewed in Bylund, 1988). All three receptor classes are expressed within the PFC, albeit at differing levels (Ramos and Arnsten, 2007; Santana et al., 2013). Adrenergic receptors appear to play an important role in working memory and attention (Coradazzi et al., 2016; reviewed in Xing et al., 2016). Of the different types of adrenergic receptors, α1A adrenergic receptors and α2A adrenergic receptors are the most studied and have been shown to be involved in working memory and top-down attentional control by the PFC (Li et al., 1999; Ramos and Arnsten, 2007; reviewed in Arnsten, 2011; Thiele and Bellgrove, 2018). α1 and α2 adrenergic receptors exert opposing effects on PFC function and working memory, with α1 adrenergic receptors impairing PFC cognitive function and α2Rs improving PFC cognitive function (Arnsten, 1997; Birnbaum et al., 1999; Ramos and Arnsten, 2007). Among α2 adrenergic receptors, evidence suggests that the α2A adrenergic receptor subtype is the densest in macaque PFC (Aoki et al., 1998b). Similarly, evidence suggests that β1 and β2 adrenergic receptors have opposing effects on PFC function and working memory, with β1 adrenergic receptors impairing working memory performance and β2 adrenergic receptors improving working memory performance, though other studies have found evidence to the contrary (Ramos et al., 2005, 2008; Ramos and Arnsten, 2007; Zhou et al., 2013). Different adrenergic receptors appear to modulate excitatory transmission in different ways. Pharmacological experiments have shown that stimulation of α1 adrenergic receptors suppresses excitatory propagation, whereas stimulation of β1 adrenergic receptors has the opposite effect (Kobayashi, 2007; Kobayashi et al., 2009). Thus, differences in the expression of the different adrenergic receptor subtypes across different cell types and layers within the FEF might have important implications for how noradrenaline affects the output of the FEF and its contribution to the control of working memory and attention.

Autoradiographic studies show that α1 and α2 adrenergic receptors are concentrated in the superficial layers of cortex, specifically layers I-IIIa, while β1 and β2 adrenergic receptors are concentrated in the intermediate layers, layers IIIb and IV (Goldman-Rakic et al., 1990). However, these studies lack cellular resolution, and may be less specific for individual receptor subtypes. Immunocytochemistry studies have characterized the sublocalization of β adrenergic receptors in the PFC on GABA-ergic interneurons (Aoki et al., 1998b). In both of these studies, however, the FEF was not among the areas surveyed. In fact, in spite of the wealth of evidence demonstrating FEF neurons contribute significantly to fundamental components of cognition, no previous work has examined the laminar and cell-type distribution of adrenergic receptors in this area of any model organism. We recently reported high levels of D1 and D2 dopamine receptor expression in pyramidal neurons, particularly long-range pyramidal neurons in the FEF (Mueller et al., 2020). Other work has shown a colocalization of D1 dopamine receptors and α1 adrenergic receptors in the PFC (Mitrano et al., 2014; reviewed in Xing et al., 2016). Thus, one might expect to observe heavy expression of α1 adrenergic receptors on long-range pyramidal neurons. There is also evidence for colocalization of D2 dopamine receptors and β1 adrenergic receptors in the PFC (Montezinho et al., 2006; reviewed in Xing et al., 2016), suggesting that β1 adrenergic receptors might be expressed more heavily on long-range pyramidal neurons as well.

We studied the pattern of adrenergic receptor expression across different layers and different classes of neurons in the FEF. Since evidence points to coexpression and cooperativity of dopamine receptors and adrenergic receptors in the PFC (Montezinho et al., 2006; Mitrano et al., 2014), we hypothesize that α1 and β1 adrenergic receptors might be expressed more heavily on long-range projecting pyramidal neurons, whereas α2 and β2 adrenergic receptors might be expressed more heavily on GABAergic interneurons, similar to patterns of dopamine expression in the FEF that have been found in previous studies (Mueller et al., 2018, 2020).

## Materials and Methods

Staining of the FEF with adrenergic receptor antibodies and cell-type-specific antibodies was carried out to identify the cell types expressing particular adrenergic receptors and to quantify the magnitude of expression within these cell types. Staining was carried out in three adult male rhesus macaque monkeys (*Macaca mulatta*). For this study, see Table 1 for specific details about the experimental history of the animals used for this study. Animals were cage-housed and periodically water-restricted for experiments in the time prior to their perfusion. Animals had daily access to environmental enrichment as well as nutritional enrichment in the form of nutritional biscuits and daily portions of fresh fruits and vegetables. Animals were routinely monitored by animal care and veterinary and laboratory staff, and also received physicals biannually in order to establish and maintain good health. All surgical procedures performed were under constant supervision by veterinary staff, and anesthetic doses and supportive medications were given and adjusted as necessary throughout the procedure. Animals were utilized for these experiments only when they reached the end-of-study time point for other experiments. All experimental procedures performed were in accordance with the NIH Guide for the Care and Use of Laboratory Animals, the Society for Neuroscience Guidelines and Policies, and the recommendations of the Stanford University Animal Care and Use Committee. The protocol was approved by the Stanford University Administrative Panel on Laboratory Animal Care.

**TABLE 1:** Animal experimental history.

Animal	Born	Age (years)	Weight (kg)	Experiments	Previous Experiments	Species	Sex
A	4/13/07	12	13.2	Immunofluorescence	Electrophysiological recordings in FEF and V4, pre-perfusion nucleation	Rhesus Macaque	Male
B	9/16/05	13	14.4	Immunofluorescence	Electrophysiological recordings in FEF, bilateral cooling loop implants in intraparietal sulcus, pre-perfusion nucleation	Rhesus Macaque	Male
C	3/26/11	8	12.5	Immunofluorescence	CT scanning, headpost and chamber implant but no craniotomy, no recordings	Rhesus Macaque	Male

### Fixation

The monkeys’ corneal and palpebral reflexes were tested (negative) prior to the perfusion and were monitored by veterinary staff both prior to and during the perfusion. Animals were anesthetized to the surgical plane with 3–4.5% isofluorane and then initially perfused with 0.25–0.5 L serological saline at high pressure. We decided not to apply a lethal injection of pentobarbital because it can result in a quicker cessation of heartbeat, and it is important that the fixative be exposed to tissue that is as healthy and oxygenated as possible to maintain quality. Then, the animals were perfused with 4 L of 3.5–4% paraformaldehyde in 0.1M phosphate buffered saline: 2 L at high pressure over 2–3 min and 2 more liters at low pressure over the course of an hour. Lastly, animals were perfused with 1 L each of 10%, 20% and 30% sucrose solutions at high pressure for cryoprotection. All perfusions were performed in the Stanford necropsy suite under ventilation. Following perfusion, the brains rested in a 30% sucrose phosphate buffered solution for 7 and 10 days. Finally, we used a freezing microtome to cut 20 μm coronal sections of the PFC and stored the sections in 0.1M phosphate buffered saline until they were ready to be stained and imaged. Animals A and B had previously been used for electrophysiological experiments, but we did not include tissue that exhibited recording track damage in our analysis. Electrophysiological recordings in these animals were made in very localized regions of cortex. In the fixed tissue, glial scarring from the tract sites made it evident which regions were damaged. We therefore quantified adrenergic receptor expression from un-damaged FEF regions adjacent to tract sites or from FEF sections with no scarring whatsoever.

### Immunofluorescence

We co-stained sections with antibodies to α1A, α2A, β1, and β2 adrenergic receptors, as well as to different neuronal markers (see Table 2). Additionally, we used western blots for all four receptor subtypes to confirm the specificity of our antibodies, incubating overnight at 4°C. We loaded either 8 or 15 μL of sample onto SDS-PAGE on 4–20% precast gels (BioRad 456-1098) and transferred to Immobilon-FL membranes (EMD Millipore IPFL00010). Membranes were blocked with Intercept Blocking Buffer (LiCor 927-70001) and then incubated overnight with anti-α1A adrenergic receptor (Alomone Labs AAR-015), anti-α2A adrenergic receptor (Alomone Labs AAR-020), anti-β1 adrenergic receptor (Alomone Labs AAR-023), or anti-β2 adrenergic receptor (Alomone Labs AAR-016) pre-incubated with peptide plus 1% BSA, or pre-incubated with 1% BSA alone. Blots were then washed in PBST (Phosphate buffered saline + 0.05% Tween 20) and incubated either with Pierce^TM^ ECL Western Blotting Substrate (Thermo Scientific 32106) or IR Dye 800CW-Donkey anti-Rabbit (LiCor 925-32213) in Intercept Blocking Buffer, washed in PBST, rinsed in PBS, and imaged using either autoradiography film or the Image Studio Lite imaging system (Li-Cor). Common negative controls for immunofluorescence experiments include demonstrating antibody specificity through the absence of primary antibody signal either after the antibody has been exposed to peptides representing a unique receptor-specific epitope or through the absence of the primary antibody itself (Leung et al., 2000; Wang et al., 2012; Pitia et al., 2015). When we pre-absorb our four adrenergic antibodies with receptor specific epitopes, immunofluorescent images of the tissue show virtually no fluorescence (Supplementary Figure 1A, middle panels). Artificially increasing the gain on these images still shows that no cells are stained with the antibody; indicating that it has been almost completely absorbed by its specific antigen-peptide (Supplementary Figure 1A, right panels). In a western blot analysis, incubation of the primary antibody with its receptor-specific antigen-peptide also caused a specific loss of staining of bands at the appropriate molecular weight for each adrenergic receptor (see Supplementary Figure 1B). Our working solution for all antibody dilutions and washes was 0.1M phosphate buffer containing 5% donkey serum (Millipore, S30-100ML) as a blocking agent. Sections were initially washed three times, then exposed to the working solution (blocking buffer) for between 1 and 1.5 h at room temperature. Then, the sections were washed three times again before being exposed to the primary antibodies at room temperature overnight, with rotation. In previous comparisons, we found that incubation at either room temperature or at 4°C made no difference to the staining quality. The following day, the sections were again washed three times and exposed to the appropriate secondary antibodies for 2 h at room temperature, with rotation. We used donkey-anti-mouse or donkey-anti-rabbit Alexa Fluor secondary antibodies in 488 and 568 wavelengths (Thermo Fisher Scientific). We used donkey anti-rabbit Alexa Fluor 488 secondary antibodies to bind to the adrenergic receptor primary antibodies (Thermo Fisher Scientific). We used donkey anti-mouse Alexa Fluor 568 secondary antibodies to bind to the primary antibodies for all of our neuronal markers. The sections were then washed another six to ten times, and then exposed to 10 mM cupric sulphate in acetate solution for 10 min in order to help quench lipofuscin particle fluorescence (Schnell et al., 1999). Finally, the sections were mounted on slides with DAPI-enriched fluoromount mounting medium (Vector Laboratories, Vectashield, H-1200). DAPI stains DNA and therefore acts as a label for all nucleated cells.

**TABLE 2:** Antibody information.

Antigen	Host	Vendor information	Dilution
α1A adrenergic receptor	Rabbit polyclonal	AAR-015; Alomone Labs, Jerusalem, Israel	1:250
α2A adrenergic receptor	Rabbit polyclonal	AAR-020; Alomone Labs, Jerusalem, Israel	1:250
β1 adrenergic receptor	Rabbit polyclonal	AAR-023; Alomone Labs, Jerusalem, Israel	1:250
β2 adrenergic receptor	Rabbit polyclonal	AAR-016; Alomone Labs, Jerusalem, Israel	1:250
Parvalbumin	Mouse monoclonal	P3088; Sigma-Aldrich Inc., St. Louis, MO	1:500
Calbindin	Mouse monoclonal	CB300: Swant Inc., Switzerland	1:500
Calretinin	Mouse monoclonal	6B3; Swant Inc., Switzerland	1:500
Neurogranin	Mouse monoclonal	SC_514992; Santa Cruz Biotechnology, Santa Cruz, CA	1:100
SMI-32	Mouse monoclonal	NE1023; Millipore, Temecula, CA	1:500
RP (rat pyramidal neurons)	Mouse monoclonal	345; Swant Inc., Switzerland	1:250
NeuN	Mouse monoclonal	ABN78; Millipore, Temecula, CA	1:1,000

### Imaging

We identified the FEF as the rostral bank of the arcuate sulcus, posterior to the principal sulcus (Moschovakis et al., 2004; Percheron et al., 2015), and we performed tile scans of continuous areas of cortex (pial surface to white matter) using a Leica TCS SP2 AOBS confocal microscope with a 20× objective. We collected confocal Z-stacks that spanned the 20 μm section depth and collapsed the resulting images across the Z-dimension for counting and illustration. At the beginning of an imaging session, we optimized laser power, gain, and offset and did not adjust these settings again thereafter. All images were taken using sequential line scans with the different laser wavelengths to reduce bleed-through. These images were then analyzed using ImageJ. All adrenergic receptors were labeled in green, and all pyramidal neurons and inhibitory interneurons were labeled in magenta.

### Quantification

Our primary assessment was the number of neurons that expressed solely adrenergic receptors, solely cell-type markers (e.g., parvalbumin, SMI-32, calbindin, etc.), and neurons that co-expressed both adrenergic receptors and cell-type markers. We counted the neurons manually using ImageJ software and the “cell counter” plugin. We manually counted neurons that expressed either adrenergic receptors, a neuronal marker, or both, on each image for each animal. High-magnification images of each of our antibody stains that demonstrates their capturing of distinct morphological identities can be found in Supplementary Figure 2. All three pyramidal neuron subtypes displayed distinct triangular (pyramidal) morphology, whereas the inhibitory interneurons displayed rounded morphology typical of interneurons. Across all animals, we used sections that included sulcal tissue in the anterior bank of the arcuate at the level of the caudal end of principalis (roughly mid-eccentric) and extending approximately 2 mm caudally. Because we used a restricted region of the FEF, we expect our tissue samples to include neurons that represent neither far foveal nor far peripheral regions. We sampled tissue sections of similar area—approximately 0.5 mm by 1.5 mm. The exact area varied because we ensured that we included all cortical layers, and cortical thickness can vary across cortex and animals. Each section was 20 μm thick and was bounded by the pial surface and white matter along one axis, a distance of 0.5 mm along the cross-axis. Although our sections were only 20 μm thick and cortical neurons approximately measure between 5 and 20 μm in diameter and rarely are physically abutting, it is theoretically possible but very unlikely that collapsing images across the Z-dimension could hide neurons that completely overlap along the *Z*-axis. Therefore, our counts are only an approximation of the true number of neurons within a particular area.

We then averaged counts across all animals, and then calculated proportions for co-expression from these across-animal averages. We estimated the number of neurons expressing a particular adrenergic receptor, neuronal marker, or both across the cortex by identifying their positions along the pia/white-matter axis. We identified the different cortical layers by visual inspection as follows: Layer I was identified as a region with very few cell bodies. Layer IV, which exists as a very narrow strip in macaque FEF (Huerta et al., 1986; Moschovakis et al., 2004; Percheron et al., 2015), was then identified as a thin band with very small, tightly packed cells. Layers II–III were therefore defined as the region located in between layers I and IV. Although we made every effort to correctly identify the border between Layer I and Layer II, because we used a single linear threshold to define the border between the two layers, it is possible that a very small fraction of neurons in Layer II could be mis-classified as residing in Layer I. Layer V was identified by the presence of large neurons with pyramidal cell morphology. Lastly, Layer VI was defined as the region located between layer V and the predominantly neuron-free white matter. We sampled tissue sections of similar area and ensured that we quantified cells across all layers of cortex so that each section included the pial surface down to the white matter.

Statistical comparisons were performed using chi-squared tests, which were then corrected for multiple comparisons using the Bonferroni method. The majority of comparisons were two-by-two: adrenergic receptor presence and absence on two different cell types, resulting in a degree of freedom of one. In order to get the data for the chi-squared tests, we performed pooled neuron counts across animals. Since our primary interest was in comparing the proportion of neurons that expressed a given receptor compared to another receptor across different subtypes, we determined that using pooled neuron counts was appropriate. Although we did not perform any statistical tests on the across-animal variability, the across-animal variability is described in Table 3 for reference, where we list the mean proportion of co-expression across animals and the associated standard error.

**TABLE 3:** Proportion of different cell types expressing adrenergic receptors α1AR, α2AR, β1R, and β2R.

	α1AR			α2AR			β1R			β2R		
				
	Mean	STE	Total N	Mean	STE	Total N	Mean	STE	Total N	Mean	STE	Total N
NRG	71.750	3.443	2,157	85.514	2.214	1,779	69.398	2.557	1,706	74.917	6.037	1,715
RP	52.543	16.375	2,806	72.814	13.465	2,152	60.886	5.109	2,821	71.926	5.812	2,843
SMI-32	95.188	2.406	358	98.198	1.802	93	97.021	2.408	260	96.600	0.986	276
Parvalbumin	61.099	4.374	587	61.699	8.446	253	53.709	4.218	520	62.610	6.834	618
Calbindin	68.934	6.538	339	77.708	4.181	382	71.879	2.462	784	84.045	2.010	617
Calretinin	54.004	9.013	606	67.662	9.575	295	25.698	4.569	461	64.432	6.370	841

**All proportions are provided as across-animal averages, and the standard error across animals is provided as well.**

## Results

We stained FEF sections for four types of adrenergic receptors: α1A, α2A, β1, and β2 adrenergic receptors as well as for three pyramidal neuron markers and four inhibitory neuron markers. Pyramidal neurons were labeled with neurogranin, a more general pyramidal neuron marker (Higo et al., 2004; Singec et al., 2004), RP, another general pyramidal neuron marker, or SMI-32, a putative marker for a subset of long-range projecting pyramidal neurons (Campbell and Morrison, 1989; Voelker et al., 2004). RP is an abbreviation of “Rat Pyramidal”, so-named because this antibody was originally developed by targeting rat pyramidal neurons. Interneurons were labeled with markers for three independent inhibitory interneuron populations, specifically parvalbumin, calbindin, and calretinin. The antibodies that we used to label the different inhibitory interneuron subtypes—parvalbumin (Lanciego and Vázquez, 2012; Thomé et al., 2016), calbindin (Lavenex et al., 2009; Timbie and Barbas, 2014), and calretinin (Lavenex et al., 2009; Bunce et al., 2013)—are established markers for their respective interneuron subtypes in primates. More information on the different antibodies that we used can be found in Table 2. We then compared coexpression of adrenergic receptors across these different populations of cells using pooled neuron counts across all animals. Chi-square tests were performed based on these pooled neuron counts. We also provide data on across-animal variability, including the across-animal averages in expression and associated standard errors, in Table 3 for reference.

### Adrenergic Receptors Are Broadly Expressed in FEF

**FIGURE 1:**
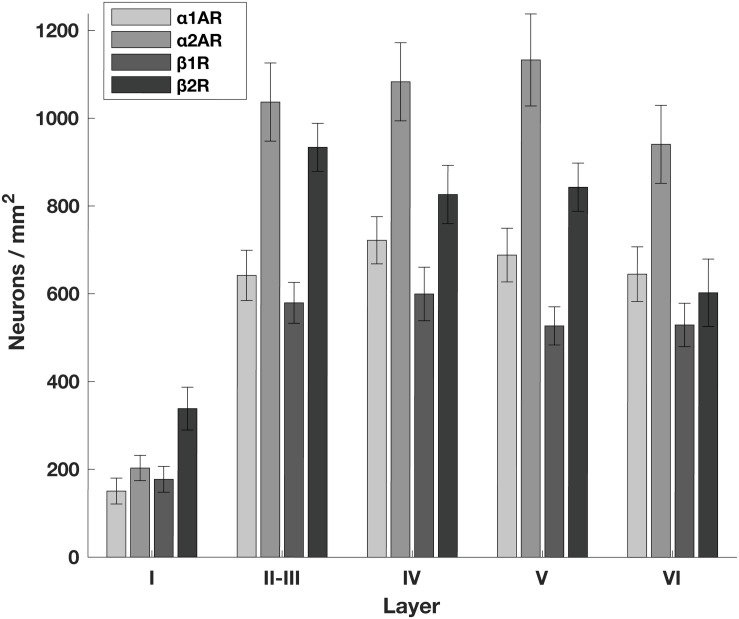
Density of adrenergic receptors across different layers of the FEF. The number of neurons per mm^2^ that express a given receptor across FEF layers. α2A adrenergic receptors (α2ARs) and β2 adrenergic receptors (β2Rs) are more abundant than either α1A adrenergic receptors (α1ARs) or β1 adrenergic receptors (β1Rs) across layers II through V. There are no obvious differences in expression across layers other than the predictably low expression of all receptor classes in layer I where there are few neurons.

**FIGURE 2:**
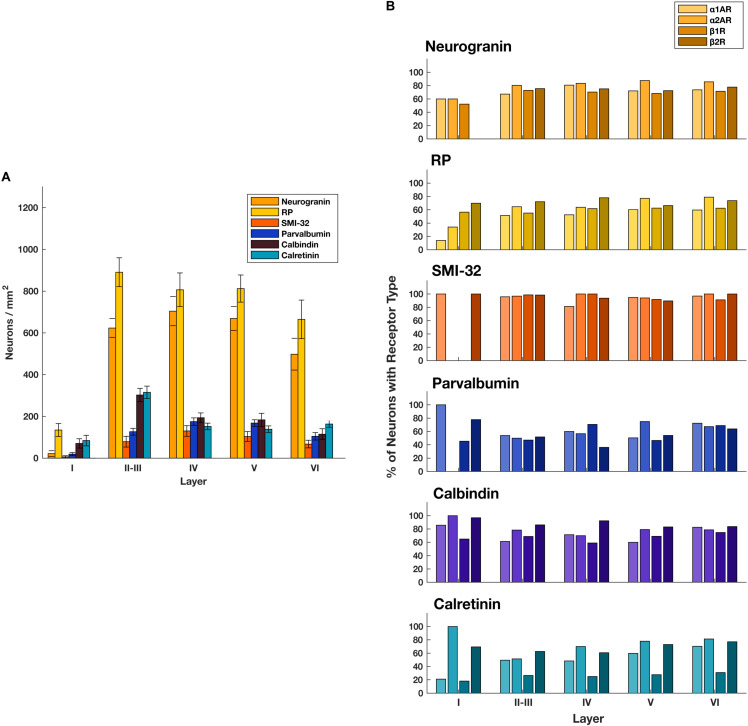
Expression of adrenergic receptors across cell types and layers. **(A)** The number of different classes of cell types per mm^2^ across FEF layers. General classes of pyramidal neurons (neurogranin and RP) are more abundant than any other class of neuron across layers II through VI. There is low expression of all receptor classes in layer I where there are few neurons. **(B)** For each of the four adrenergic receptors (pale to dark: α1A, α2A, β1, β2) we quantified the proportion of each cell type that expressed that receptor across all cortical layers in the FEF. We found that expression was very consistent for any given receptor/cell type pair.

Generally, we found that α2A adrenergic receptors and β2 adrenergic receptors were more abundant than either α1A adrenergic receptors or β1 adrenergic receptors across layers II through V. We found no obvious differences in across-layer expression within any adrenergic receptor subtype, aside from the predictably low expression of all receptor classes in layer I where there are few neurons (Figure 1). We found a significant difference in the density of receptors across different receptor types (*p* < 10^–19^, 3 d.f.) as well as a significant difference in the density of receptors across different layers (*p* < 10^–40^, 4 d.f.). In addition, neurogranin+ and RP+ general pyramidal neurons were more abundant than other cell types across layers II through VI (Figure 2A). Generally, we observed that all four adrenergic receptors were expressed in roughly equal proportions across layers II through VI among all neuronal subtypes. One exception to this observation was the expression of β1 adrenergic receptors in calretinin+ neurons, which was lower than the expression of the other three adrenergic receptor subtypes across all layers (Figure 2B). Condé et al. (1994) find that in Area 46 of macaque PFC, calretinin and calbindin expression is higher in layers II–III compared to deeper layers IV–VI. This is consistent with our results where we show a significantly higher density of calretinin+ and calbindin+ neurons in layers II–III compared to layers IV–VI (Figure 2A). Regarding parvalbumin expression, studies in macaque V1, macaque MT, and rat frontal cortex (FC) suggest that parvalbumin is expressed in roughly equal proportions across layers II–VI, which is also what we see in our study (Figure 2A). Disney and Aoki (2008) find that “PV−ir neurons appear evenly distributed throughout layers 2–6” in macaque V1. Similarly, Disney et al. (2014) find that “parvalbumin (PV) neurons are present in cortical layers 2 through 6 in both areas (V1 and MT)” and appear to be roughly evenly distributed. Kubota et al. (1994) find that parvalbumin is expressed at a very similar level in layers II-III compared to layer V of rat frontal cortex (FC), with a density of 10 cells/0.1 mm^2^ and 9.4 cells/0.1 mm^2^, respectively. Similarly, Xu et al. (2010) also find that parvalbumin (PV) is expressed at a very similar level across layers II-V of rat FC: PV density is 215 cells/mm^2^ in layer II-III, 242.6 cells/mm^2^ in Layer IV, and 226.6 cells/mm^2^ in layer V.

**FIGURE 3:**
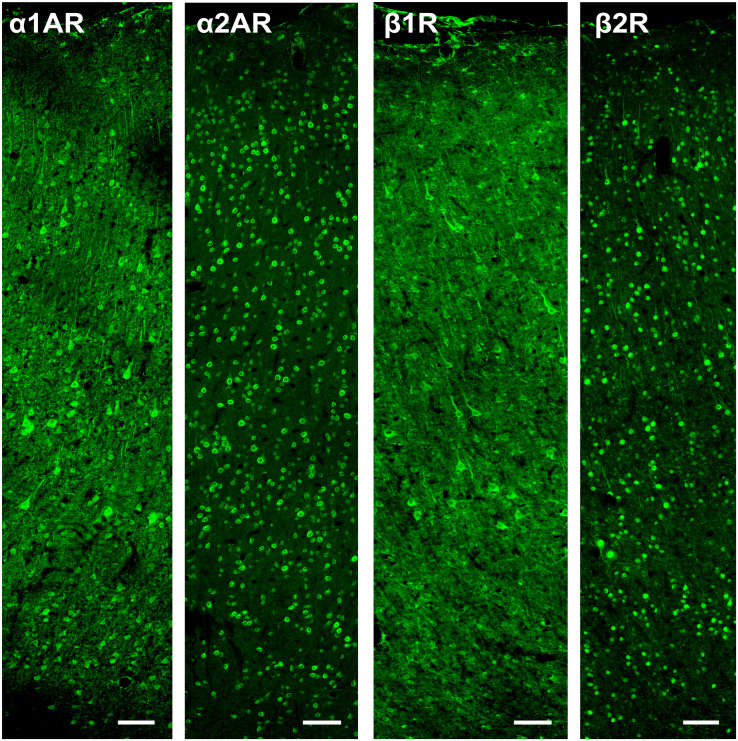
Expression of adrenergic receptors in FEF. From left to right: expression of α1A, α2A, β1, and β2 adrenergic receptors (α1AR, α2AR, β1R, and β2R, respectively) in macaque FEF. Images show a cross-section of all layers of cortex and are oriented with the pial surface at the top and white matter at the bottom. The α2A and β2 adrenergic receptors had strong, punctate staining of cell bodies, with little to no background labeling of processes. While the α1A and β1 adrenergic receptors also had strong, punctate staining of cell bodies, there was also staining of the surrounding processes (dendrites and axons), which resulted in a higher amount of background signal. Scale bar = 100 μm for all panels.

**FIGURE 4:**
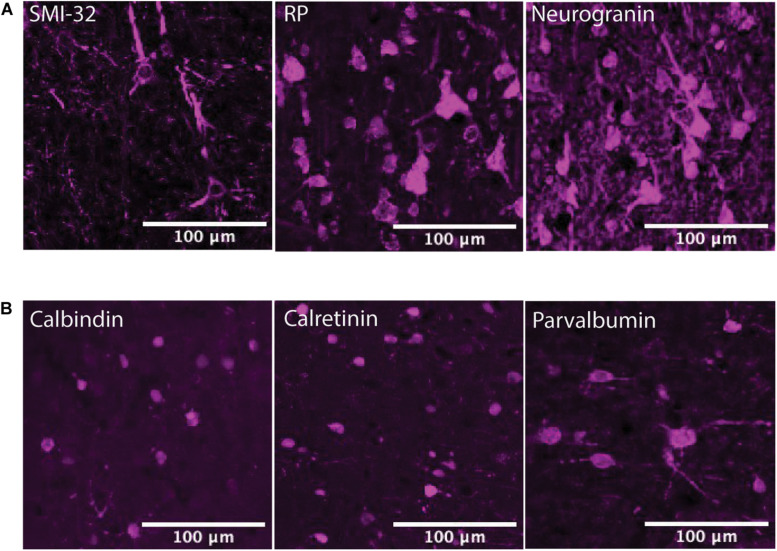
Pyramidal neuron and interneuron morphology. **(A)** Panels show expression of SMI-32+ pyramidal neurons, RP+ pyramidal neurons, and neurogranin+ pyramidal neurons in macaque FEF, from left to right. All three subtypes of pyramidal neurons display distinct triangular (pyramidal) morphology. Scale bar = 100 μm for all panels. **(B)** Panels show expression of calbindin+ interneurons, calretinin+ interneurons, and parvalbumin+ interneurons in macaque FEF, from left to right. All three subtypes of inhibitory interneurons display rounded morphology typical of interneurons. Scale bar = 100 μm for all panels.

The α2A and β2 adrenergic receptor labeling exhibited strong, punctate staining of cell bodies, with little to no background labeling of processes. On the other hand, while the α1A and β1 adrenergic receptor labeling also exhibited strong, punctate staining of cell bodies, the surrounding processes (dendrites and axons) were also stained. This resulted in a higher amount of background signal (Figure 3). Figure 4 shows higher magnification images of each of our antibody stains that demonstrate their capturing of distinct morphological identities. All three pyramidal neurons display distinct triangular morphology typical of pyramidal neurons, whereas the inhibitory interneurons display rounded morphology typical of interneurons.

### Adrenergic Receptor Expression Among Putative Long-Range Projecting Pyramidal Neurons Is Higher Than Among General Pyramidal Neurons

**FIGURE 5:**
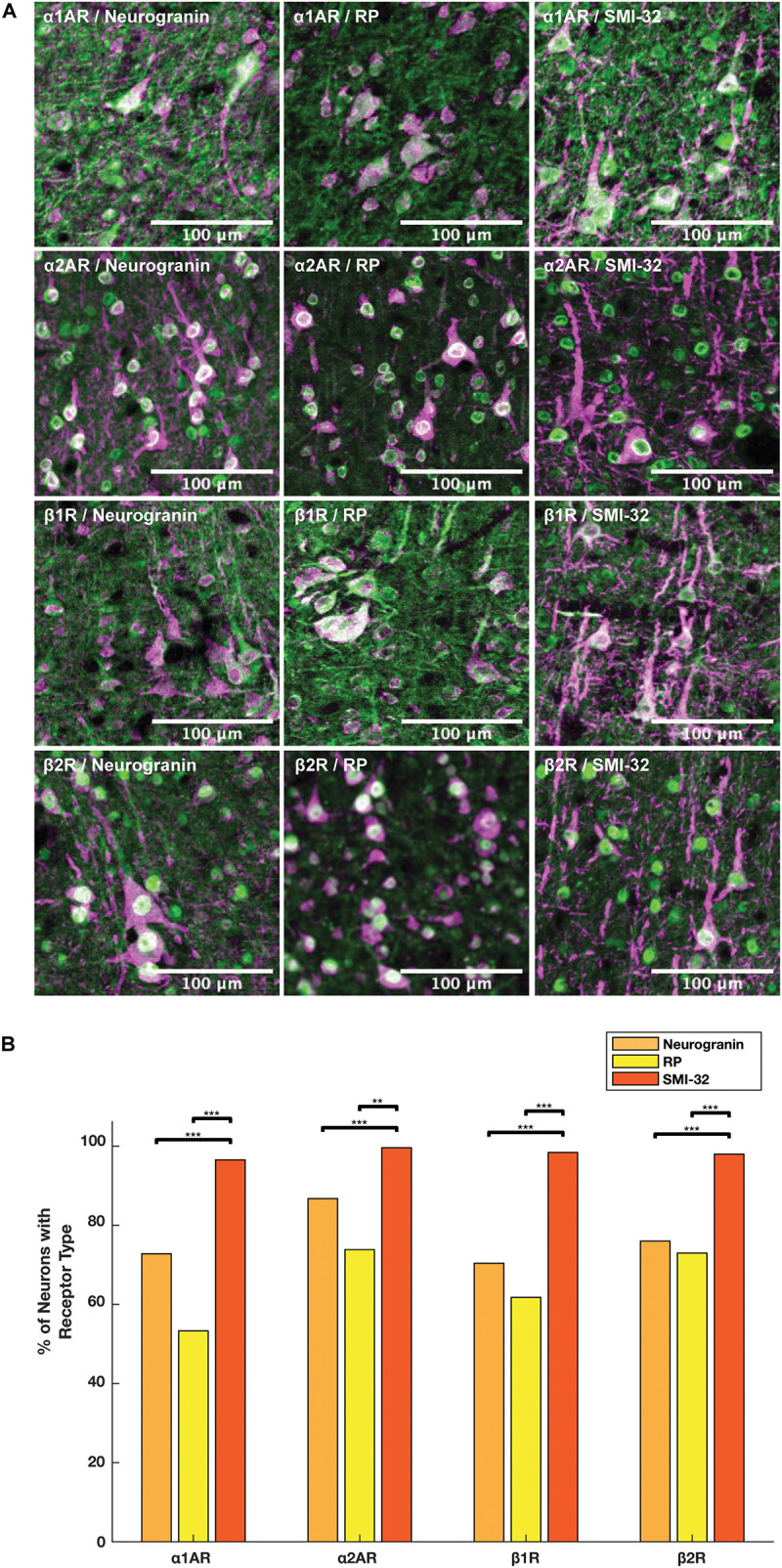
Expression of adrenergic receptors on pyramidal neurons. **(A)** Panels show expression of α1A, α2A, β1, and β2 adrenergic receptors (α1AR, α2AR, β1R, and β2R, respectively) from top to bottom with pyramidal neuron markers (RP, neurogranin, and SMI-32) from left to right. RP and neurogranin are both putative general markers of pyramidal neurons and SMI-32 is a marker for putative long-range projecting pyramidal neurons. All adrenergic receptors are labeled in green, and all pyramidal neurons are labeled in magenta. **(B)** Quantification of the proportion of each neuron class that expressed each receptor class. Chi-squared tests were performed using pooled neuron counts across all animals. All four adrenergic receptors were expressed significantly more highly on long-range projecting pyramidal neurons than either class of general pyramidal neuron. Significance levels are noted as ***p < 0.001; **p < 0.01; and *p < 0.05. Scale bar = 100 μm for all panels.

We compared the prevalence of α1A, α2A, β1, and β2 adrenergic receptors on SMI-32+, neurogranin+, and RP+ pyramidal neurons (Figure 5A). In order to perform these comparisons, we used pooled neuron counts across all animals. Chi-squared tests to compare proportions were performed using these pooled neuron counts. We observed that adrenergic receptor expression among SMI-32+ long-range projecting pyramidal neurons was much higher than adrenergic receptor expression on either class of general pyramidal neuron (neurogranin+ and RP+) (Figure 5B). In fact, virtually all SMI-32+ long-range projecting pyramidal neurons expressed all four classes of adrenergic receptors. α1A adrenergic receptors were expressed at a significantly higher rate (95.2%) on SMI-32+ long-range projecting pyramidal neurons than on neurogranin+ and RP+ general pyramidal neurons (71.8%, *p* < 10^–16^ and 52.5%, *p* < 10^–16^, respectively). Similar results were found for α2A adrenergic receptors (98.2%, SMI-32+ neurons; 85.5% neurogranin+ neurons, *p* < 10^–3^; 72.8% RP+ neurons, *p* < 10^–8^), for β1 adrenergic receptors (97.0% SMI-32+ neurons; 69.4% neurogranin+ neurons, *p* < 10^–15^; 60.9% RP+ neurons, *p* < 10^–16^), and for β2 adrenergic receptors (96.6% SMI-32+ neurons; 74.9% neurogranin+ neurons, *p* < 10^–14^; 71.9% RP+ neurons, *p* < 10^–16^; Tables 3, 4). Overall, we found that there were statistically significant differences in adrenergic receptor expression when comparing SMI-32+ neurons and neurogranin+ neurons, as well as when comparing SMI-32+ neurons and RP+ neurons, across all four adrenergic receptor subtypes tested. There were no statistically significant differences between adrenergic receptor expression on neurogranin+ and RP+ general pyramidal neurons among any of the four adrenergic receptor subtypes tested (Figure 5B).

**TABLE 4:** *P*-values of pairwise comparisons for adrenergic receptor expression across pyramidal neurons.

	Neurogranin vs. SMI-32	RP vs. SMI-32
α1AR	**<10**^–^**^16^**	**<10**^–^**^16^**
α2AR	**4.581 × 10**^–^**^4^**	**2.970 × 10**^–^**^9^**
β1R	**1.110 × 10**^–^**^16^**	**<10**^–^**^16^**
β2R	**2.998 × 10**^–^**^15^**	**<10**^–^**^16^**

**p = 0.00625 with Bonferroni correction for 8 comparisons is equivalent to the 0.05 threshold of uncorrected statistical significance. Bolded p-values are significant at the 0.05 threshold of uncorrected statistical significance.**

### Adrenergic Receptors Are Expressed in Different Proportions Among Calbindin+ and Calretinin+ Inhibitory Interneurons

**FIGURE 6:**
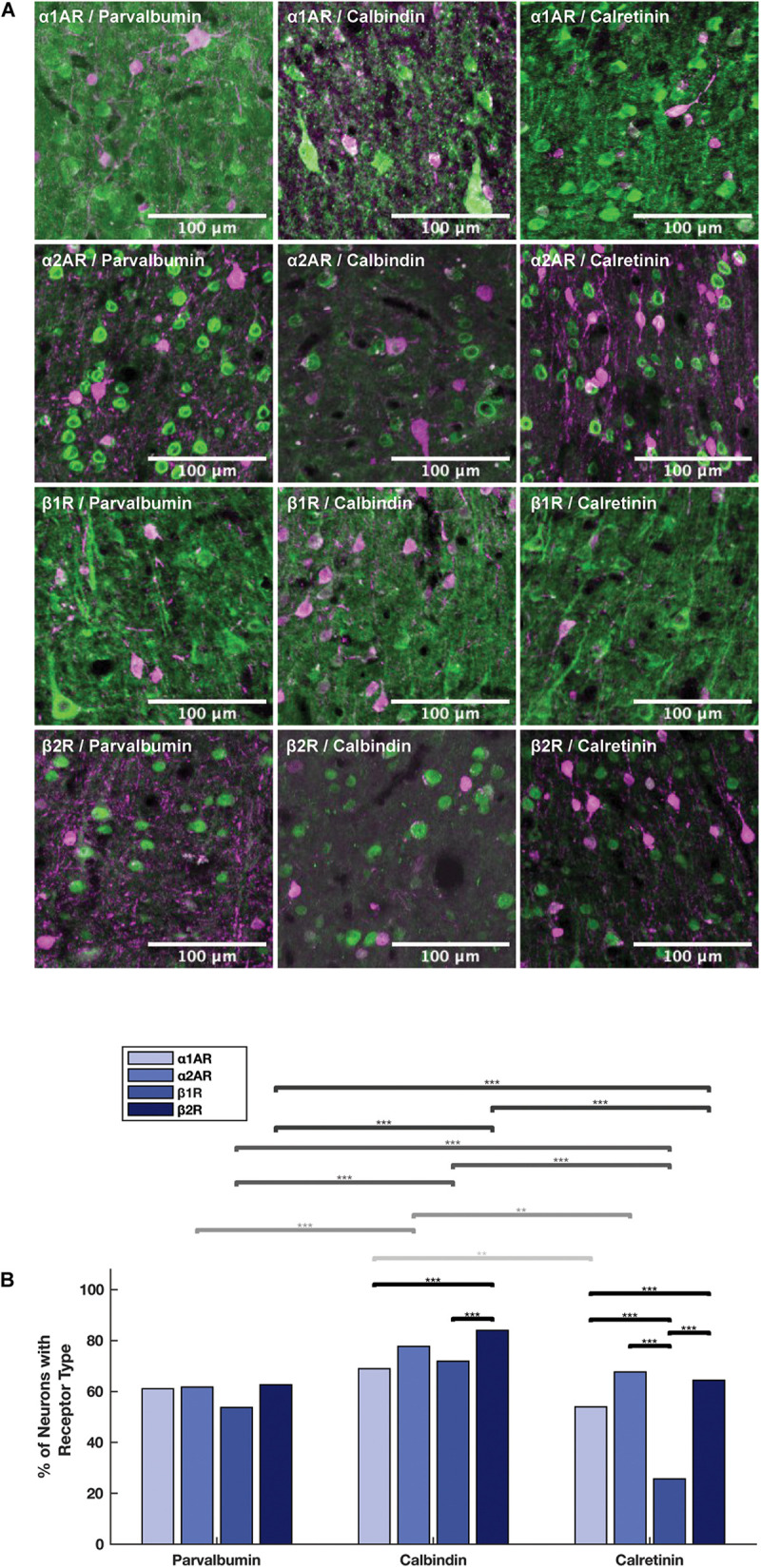
Expression of adrenergic receptors on inhibitory interneurons. **(A)** Panels show expression of α1A, α2A, β1, and β2 adrenergic receptors (α1AR, α2AR, β1R, and β2R, respectively) from top to bottom with inhibitory interneuron markers (parvalbumin, calbindin and calretinin) from left to right. All adrenergic receptors are labeled in green, and all inhibitory interneurons are labeled in magenta. **(B)** Quantification of the proportion of each neuron class that expressed each receptor class. Chi-squared tests were performed using pooled neuron counts across all animals. Lines above the bars show the significance of different comparisons. Black lines indicate significant differences between the expression of different receptors within a neuron class; gray lines indicate significant differences of expression of a specific receptor across different neuron classes. The shade of gray indicates which receptor class is being compared and matches the shading of the bars: from light to dark—α1AR, α2AR, β1R, and β2R. Significance levels are noted as ***p < 0.001; **p < 0.01; and *p < 0.05. Scale bar = 100 μm for all panels.

We examined adrenergic receptor expression among parvalbumin+, calbindin+, and calretinin+ inhibitory interneurons (Figure 6A). In order to perform these comparisons, we used pooled neuron counts across all animals. Chi-squared tests were performed based on these pooled neuron counts. Within parvalbumin+ interneurons, expression of all four adrenergic receptor subtypes was roughly the same. Although we found a slightly lower level of expression of β1 adrenergic receptors (53.7%) as compared to α1A adrenergic receptors (61.1%), α2A adrenergic receptors (61.7%), and β2 adrenergic receptors (62.6%) (Table 3) in parvalbumin+ interneurons, none of the *p*-values are statistically significant (Figure 6B). Within calbindin+ interneurons, expression of β2 adrenergic receptors (84.0%) was significantly higher than that of β1 adrenergic receptors (71.9%, *p* < 10^–9^) and α1A adrenergic receptors (68.9%, *p* < 10^–8^) (Tables 3, 5). Furthermore, expression of adrenergic receptors differed significantly across calretinin+ interneurons: each class of receptor was expressed at a statistically significantly different level than every other class of receptor, with the exception of the difference in expression of α1A and α2A adrenergic receptors and the difference in expression between β1 and β2 adrenergic receptors (Figure 6B and Table 5). Within calretinin+ interneurons in particular, we found much lower levels of expression of β1 adrenergic receptors (25.7%) than of α1A (54.0%), α2A (67.7%), and β2 (64.4%) adrenergic receptors (Table 5).

**TABLE 5:** *P*-values of pairwise comparisons for adrenergic receptor expression within inhibitory interneurons.

	α1AR vs. α2AR	α1AR vs. β1R	α1AR vs. β2R	α2AR vs. β1R	α2AR vs. β2R	β1R vs. β2R
Parvalbumin	0.355	0.006	0.022	0.213	0.399	0.584
Calbindin	0.005	0.539	**5.849 × 10**^–^**^9^**	0.007	0.007	**4.619 × 10**^–^**^10^**
Calretinin	0.007	**<10^–16^**	**3.527 × 10^–7^**	**<10^–16^**	0.206	**<10**^–^**^16^**

**p = 0.0028 with Bonferroni correction for 18 comparisons is equivalent to the 0.05 threshold of uncorrected statistical significance. Bolded p-values are significant at the 0.05 threshold of uncorrected statistical significance.**

### Adrenergic Receptors Are Expressed in Higher Proportions on Calbindin+ Interneurons Than Calretinin+ and Parvalbumin+ Interneurons

Having examined the expression of adrenergic receptors within the different types of interneurons, we next compared the expression of adrenergic receptors across the different interneuron types. In order to perform these comparisons, we used pooled neuron counts across all animals. Chi-square tests were performed based on these pooled neuron counts. We found that the expression of all four adrenergic receptors was higher in calbindin+ interneurons than both calretinin+ and parvalbumin+ interneurons (Figure 6B), and almost all differences were found to be statistically significant, with the exception of the difference in α1A adrenergic receptor expression among calbindin+ interneurons vs. among parvalbumin+ interneurons (Table 6). The biggest differences in adrenergic receptor expression between the different types of interneurons were found in β1 adrenergic receptors, which were expressed in 71.9% of calbindin+ interneurons as compared to only 25.7% of calretinin+ interneurons (*p* < 10^–16^) and 53.7% of parvalbumin+ interneurons (*p* < 10^–7^), and β2 adrenergic receptors, which were expressed in 84.0% of calbindin+ interneurons as compared to only 64.4% of calretinin+ interneurons (*p* < 10^–10^) and 62.6% of parvalbumin+ interneurons (*p* < 10^–16^) (Tables 3, 6).

**TABLE 6:** *P*-values of pairwise comparisons for adrenergic receptor expression across inhibitory interneurons.

	Parvalbumin vs. Calbindin	Parvalbumin vs. Calretinin	Calbindin vs. Calretinin
α1AR	0.095	0.012	**1.473 × 10**^–^**^4^**
α2AR	**9.243 × 10**^–^**^7^**	0.176	**3.085 × 10**^–^**^4^**
β1R	**1.385 × 10**^–^**^8^**	**< 10**^–^**^16^**	**<10**^–^**^16^**
β2R	**<10**^–^**^16^**	**1.113 × 10**^–^**^6^**	**6.133 × 10**^–^**^11^**

**p = 0.0042 with Bonferroni correction for 12 comparisons is equivalent to the 0.05 threshold of uncorrected statistical significance. Bolded p-values are significant at the 0.05 threshold of uncorrected statistical significance.**

### SMI-32+ Pyramidal Neurons Have Higher Levels of Adrenergic Receptor Expression Than All Other Neuron Subtypes

Overall, we found that expression of adrenergic receptors among SMI-32+ putative long-range pyramidal neurons was highest compared to all neuron subtypes tested (both interneurons and pyramidal neurons). In fact, almost all SMI-32+ long-range pyramidal neurons expressed all four types of adrenergic receptors (Table 3). This observation was consistent across all layers except for layer I (Figure 2B).

## Discussion

We observed that all four types of adrenergic receptors are broadly expressed across layers II to VI of FEF. Importantly, we found that among pyramidal neurons, for all four receptor classes, a significantly higher proportion of adrenergic receptors are expressed on SMI-32+ putative long-range projecting pyramidal neurons than either neurogranin+ or RP+ general pyramidal neurons. Finally, we found that there were cell-type specific differences in adrenergic receptor expression within calbindin+ and calretinin+ interneurons, as well as differences in expression across different interneuron subtypes, with higher levels of adrenergic receptor expression on calbindin+ interneurons compared to both calretinin+ and parvalbumin+ interneurons.

### Adrenergic Receptors Are Disproportionately Expressed on Long-Range Projecting Pyramidal Neurons

We found that SMI-32+ long-range projecting pyramidal neurons expressed adrenergic receptors in a higher proportion than either neurogranin+ or RP+ general pyramidal neuron classes across all four adrenergic receptor subtypes. The disproportionately high rate of adrenergic receptor expression among long-range projecting pyramidal neurons mirrors what was found for dopamine receptors in macaque FEF (Mueller et al., 2018, 2020). However, unlike dopamine receptors, which had higher rates of expression of D1Rs compared to D2Rs (Mueller et al., 2020), all four adrenergic receptor subtypes are expressed in very similar proportions on long-range pyramidal neurons. Although dopamine has been shown to modulate the influence of FEF neurons on visual signals (Noudoost and Moore, 2011a; Mueller et al., 2020), the role of noradrenaline is much less-understood. It is possible that noradrenaline is more associated with the modulation of global arousal, as opposed to selective visual attention (Berridge and Waterhouse, 2003; Noudoost and Moore, 2011b). Yet α1A and α2A adrenergic receptors, which are the most well-studied, are both thought to play roles in working memory and top-down control in the PFC (Li et al., 1999; Ramos and Arnsten, 2007; reviewed in Arnsten, 2011; Thiele and Bellgrove, 2018).

Long-range pyramidal projections from FEF to visual cortex are hypothesized to shape attention-related modulation of visual activity (Moore and Armstrong, 2003; Ekstrom et al., 2008; Zhou and Desimone, 2011; Gregoriou et al., 2012). Many different models of PFC function, such as attention and working memory, rely on persistent activity generated by the recurrent connectivity of pyramidal neurons (Wang, 1999; Durstewitz et al., 2000; Deco and Rolls, 2003; Riley et al., 2017). Whereas some studies in the literature suggest that activation of α1A adrenergic receptors and α2A adrenergic receptors have opposing effects on PFC function when it comes to working memory (Ramos and Arnsten, 2007), other studies provide evidence that persistent activity among adrenergic receptors in the PFC is mediated through a synergistic relationship between α1A adrenergic receptors and α2A adrenergic receptors of PFC pyramidal neurons (Zhang et al., 2013).

Previous work suggests that α1A adrenergic receptors help enhance glutamate release to induce persistent firing activity in the PFC, and that α2A adrenergic receptors inhibit hyperpolarization-activated cyclic nucleotide-gated (HCN) cation channels to help facilitate NE-induced persistent firing activity by α1A adrenergic receptors (Arnsten, 2011; Zhang et al., 2013). Our finding that all four classes of adrenergic receptors that we examined were predominantly expressed on long range projecting pyramidal neurons is consistent with the hypothesized synergistic relationship between α1A and α2A adrenergic receptors. Furthermore, β1 and β2 adrenergic receptors might similarly modulate visual cortical activity by acting directly on long-range projecting pyramidal neurons in the FEF.

### Adrenergic Receptor Expression Patterns Among Inhibitory Interneurons in the PFC Are Similar Across Different Species

This is the first systematic examination of adrenergic receptor expression on different cell types in macaque FEF. Though there have been limited studies characterizing adrenergic receptor expression on GABAergic interneurons in the PFC (He et al., 2014; Liu et al., 2014), these studies have been performed almost exclusively in mice, and there is a gap in the literature regarding adrenergic receptor expression on pyramidal neurons in the PFC. Furthermore, no other studies to date have characterized adrenergic receptor expression in specifically the FEF in any species.

Previous results demonstrate that within mouse PFC, α2A adrenergic receptors are highly expressed in both parvalbumin+ and calretinin+ interneurons across all layers of PFC, with different layers (Layer 2, 3 and Layer 5, 6) expressing roughly equal proportions of both parvalbumin and calretinin (He et al., 2014). This is consistent with what we found in our results—adrenergic receptor expression in parvalbumin+ and calretinin+ interneurons are roughly equal (and less than calbindin+ interneurons), and they are broadly expressed across all layers. A similar study that was performed on β1 and β2 adrenergic receptors in the PFC found that relative to parvalbumin+ and calbindin+ interneurons, calretinin+ interneurons are less likely to express β1 and β2 adrenergic receptors. This same study also found that β1 and β2 adrenergic receptors were expressed in roughly equal proportions both within and across parvalbumin+ and calbindin+ interneurons (Liu et al., 2014). Our results for β1 adrenergic receptors across cell types were consistent with other studies—we found very low levels of β1 adrenergic receptor expression relative to the other adrenergic receptor subtypes on calretinin+ interneurons in particular. We also found that β1 and β2 adrenergic receptors were expressed in similar proportions both within and across parvalbumin+ and calbindin+ interneurons. However, our results for β2 adrenergic receptors differed slightly from other studies: we found that calbindin+ interneurons had slightly higher expression of β2 than calretinin+ interneurons, which in turn had slightly higher levels of β2 expression than parvalbumin+ interneurons (Figure 6B). We found that adrenergic receptors are expressed in slightly different proportions on both calbindin+ and calretinin+ interneurons, but are expressed in similar proportions on parvalbumin+ interneurons (Table 3). Santana et al. (2013) find that GABAergic interneurons in Layers II–III and Layer V of the dorsal anterior cingulate and prelimbic areas of rat mPFC express α1A adrenergic receptors in roughly equal proportions (between 72 and 79%, Table 1 of their report), which are similar to expression levels for the other three adrenergic receptor subtypes within different interneuron subtypes found in He et al. (2014) and Liu et al. (2014). Overall, our findings that several adrenergic receptor subtypes are expressed by multiple interneuron classes in roughly similar levels are consistent with the literature describing adrenergic receptor expression in other areas and species. These findings suggest that receptor-specific adrenergic modulation in the FEF is mediated by calretinin+ and calbindin+ interneurons rather than parvalbumin+ interneurons.

### Implications for Cooperative Modulation Between Adrenergic and Dopaminergic Receptors Within the FEF Microcircuit

Research in rodents suggests that monoamine receptors (serotonergic, dopaminergic, and adrenergic receptors) are present in all layers of the PFC on both pyramidal neurons as well as GABAergic interneurons, and are involved in shaping attention and working memory activity through the modulation of excitatory inputs and the control of local microcircuits (Robbins and Arnsten, 2009; Santana and Artigas, 2017). We found similarities in expression of adrenergic receptors to dopaminergic D1, D2, and D5 (Mueller et al., 2018, 2020) receptors in different FEF cell types: namely that adrenergic receptors are much more heavily expressed in long-range pyramidal neurons compared to pyramidal neurons in general. This suggests that neuromodulators exert their influence heavily, and non-specifically, on the output of the FEF. Past work on the role of FEF neurons in visual spatial attention suggests that extrastriate cortex-projecting FEF neurons influence visual cortical activity in conjunction with eye movement preparation (Moore and Armstrong, 2003; Gregoriou et al., 2012). Thus, neuromodulation of long-range projecting neurons may be well-positioned to mediate the FEF’s control of visual attention.

More recent work shows that visual-cortex projecting FEF neurons disproportionately exhibit working memory-related activity (Merrikhi et al., 2017). It is thus noteworthy that adrenergic modulation in the PFC exhibits an inverted-U like influence on working memory performance. That is: both very low and very high levels of adrenergic signaling can lead to impaired working memory (Robbins and Arnsten, 2009; Berridge and Spencer, 2016; Datta et al., 2019). Dopamine receptor modulation exhibits a similar effect in the PFC: both very low and very high dopamine receptor activation lead to decreases in performance on different types of working memory tasks (Vijayraghavan et al., 2007; Cools and D’Esposito, 2011; Dent and Neill, 2012). Similar expression patterns between adrenergic receptors and dopamine receptors on both long-range pyramidal neurons as well as inhibitory interneurons (Mueller et al., 2018, 2020) support existing data showing that dopaminergic and adrenergic receptors colocalize on presynaptic and postsynaptic neurons to cooperatively regulate PFC activity in certain pathways (Xing et al., 2016). Mechanistic models of attentional control posit that working memory signals within PFC are used to deploy attentional selection to memorized locations and other stimulus features (Knudsen, 2007), and the interdependence of attention and working memory is well-documented (Jonikaitis and Moore, 2019). Collectively, the pattern of dopaminergic and adrenergic expression within PFC, and specifically within the FEF, suggests a means by which neuromodulators exert control on both attention and working memory. Future work might seek to examine the co-expression of dopamine and noradrenaline receptors on PFC neurons to further understand the common and distinct influences these two neuromodulators have on basic cognitive functions.

## Data Availability Statement

The raw data supporting the conclusions of this article will be made available by the authors, without undue reservation, to any qualified researcher.

## Ethics Statement

The animal study was reviewed and approved by the Stanford University Animal Care and Use Committee.

## Author Contributions

ML, AM, and TM contributed to the conception and design of the study. ML and AM drafted and wrote the manuscript, performed the experiments, and analyzed the data. TM critically revised the manuscript. All authors contributed to the revision of the manuscript and approved the final version of the submitted manuscript.

## Conflict of Interest

The authors declare that the research was conducted in the absence of any commercial or financial relationships that could be construed as a potential conflict of interest.
