# Supplementary material for: Differences in Noradrenaline Receptor Expression Across Different Neuronal Subtypes in Macaque Frontal Eye Field
Source: Front Neuroanat. 2020 Nov 26;14:574130. doi: 10.3389/fnana.2020.574130 (PMC7732642; doi:10.3389/fnana.2020.574130)
Supplement: Supplementary file 1 [file Table_1.docx]

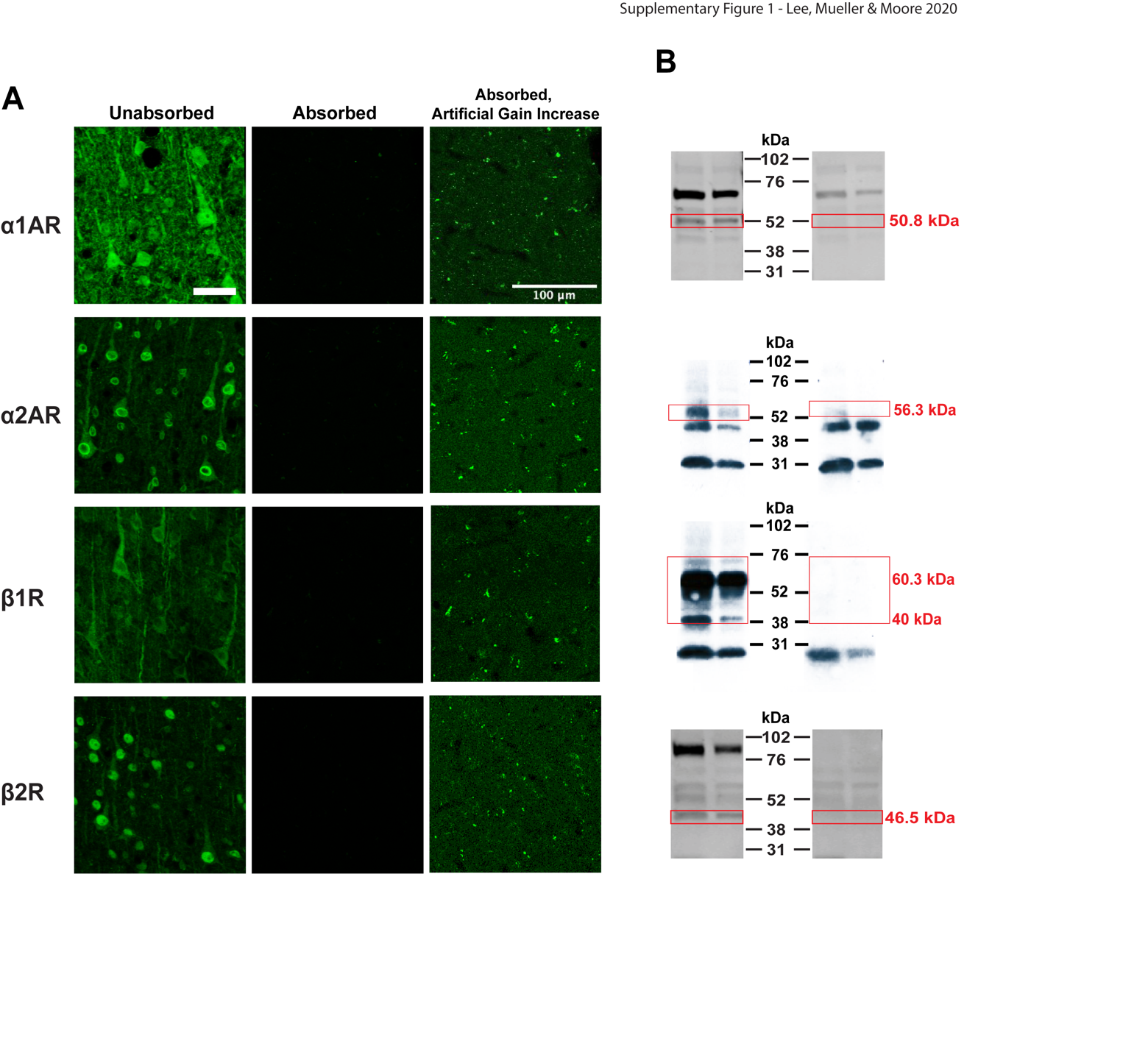


**Supplementary Figure 1**: Primary Antibody Controls

**A:** We find that for all antibodies (to all four adrenergic receptor subtypes: α1ARs, α2ARs, β1Rs, and β2Rs), incubation of the primary antibody with a peptide that represents a unique epitope specific to these receptors causes a loss of staining. The antibodies are absorbed by the peptide and we therefore see a lack of immunofluorescence in the sections that were exposed to the absorbed antibody compared to the regular, unabsorbed sections. Artificially increasing the gain on these images still shows that no cells are stained with the antibody; indicating that it has been almost completely absorbed by its specific antigen-peptide. Scale bar = 50 μm and is the same for all panels. **B:** Western blots validating antibodies used for immunofluorescent studies in A. From top to bottom: α1ARs, α2ARs, β1Rs, and β2Rs, respectively. Each antibody is tested at two dilutions of the blood serum sample. Antibodies were used unabsorbed (left) and pre-absorbed with an appropriate blocking peptide (right). For each tested antibody, the electrophoretic bands that corresponded to the predicted size of a receptor were detected (red rectangles in “Unabsorbed” column), and disappeared when the antibody is incubated with a blocking peptide prior to blotting (red rectangles in “Absorbed” column). Predicted protein sizes (Uniprot.org) are 50.8 kDa, 50.8 kDa, 51.6 kDa, and 46.5 kDa, for α1ARs, α2ARs, β1Rs, and β2Rs, respectively. In all cases the two bands represent two different volumes of loaded substrate: 15 µL, left and 8 µL, right, respectively. The bands were identified by calculation of molecular weights from a standard marker using Fiji. We used this marker between every two lanes of the gel. The results of the westerns are consistent with nonspecific labeling of several denatured proteins, but specific labeling of our adrenergic receptor specific proteins. All four western blot comparisons show a specific loss of bands representing a unique epitope corresponding to their respective receptors when the antibody was pre-absorbed with its specific antigen.
